# Dietary proanthocyanidins modulate BMAL1 acetylation, Nampt expression and NAD levels in rat liver

**DOI:** 10.1038/srep10954

**Published:** 2015-06-08

**Authors:** Aleix Ribas-Latre, Laura Baselga-Escudero, Ester Casanova, Anna Arola-Arnal, M-Josepa Salvadó, Cinta Bladé, Lluís Arola

**Affiliations:** 1 Nutrigenomic Research Group. Department of Biochemistry and Biotechnology, Universitat Rovira i Virgili, Tarragona, Spain

## Abstract

Metabolism follows circadian rhythms, which are driven by peripheral clocks. Clock genes in the liver are entrained by daytime meals and food components. Proanthocyanidins (PAs), the most abundant flavonoids in the human diet, modulate lipid and glucose metabolism. The aim of this study was to determine whether PAs could adjust the clock system in the liver. Male Wistar rats were orally gavaged with 250 mg grape seed proanthocyanidin extract (GSPE)/kg body weight at zeitgeber time (ZT) 0 (light turned on), at ZT12 (light turned off), or before a 6 hour jet-lag and sacrificed at different times. The 24 hour rhythm of clock-core and clock-controlled gene expression indicated that nicotinamide phosphoribosyltransferase (Nampt) was the most sensitive gene to GSPE. However, Nampt was repressed or overexpressed after GSPE administration at ZT0 or ZT12, respectively. NAD levels, which are controlled by Nampt and also exhibit circadian rhythm, decreased or increased according to Nampt expression. Moreover, the ratio of acetylated Bmal1, that directly drives Nampt expression, only increased when GSPE was administered at ZT12. Therefore, GSPE modulated the clock system in the liver, suggesting that PAs can regulate lipid and glucose metabolism by adjusting the circadian rhythm in the liver.

Digestion, absorption and metabolism follow circadian rhythms that are regulated by peripheral clocks^1^, and the disruption of the clock system triggers different types of illnesses, indicating that peripheral clocks play important roles in maintaining homeostasis and normal body function^2^. In this way, association studies have revealed that shift workers, night workers, and sleep-deprived individuals (clear examples of disrupted circadian rhythms) have an increased risk of developing metabolic syndrome^3^,^4^.

Among peripheral clocks, the clock system in the liver is one of the most important, because this organ plays a central role in metabolism and energy production, thus significantly affecting the physiological status of the whole organism. For instance, the liver is the major site of intermediate metabolism, including the synthesis and removal of cholesterol^5^, as well as the regulation of glucose homeostasis^6^. In fact, 10% of all transcripts, or 20% of all proteins, in mouse liver are under circadian regulation^7^, underscoring the importance of the clock present in this organ.

At the molecular level, the clock system consists of transcription–translation autoregulatory feedback loops. Driving the positive side of this loop are the transcriptional activators circadian locomotor output cycles kaput (CLOCK) and brain and muscle ARNT-like protein 1 (BMAL1). After forming a heterodimer, these factors activate the transcription of the Period (*Per*) and Cryptochrome (*Cry*) genes. In turn, once they reach a critical concentration, PER and CRY proteins translocate to the nucleus and inhibit the activity of the CLOCK:BMAL1 heterodimer. In addition, the active CLOCK:BMAL1 heterodimer also promotes the transcription of retinoic acid-related orphan receptor alpha (*Rorα*) and nuclear receptor subfamily 1, group D, member 1 (*Nr1d1*, also known as *Rev-erbα*), its own activator and repressor, respectively, generating another loop of regulation. Finally, the CLOCK:BMAL1 heterodimer enhances the transcription of metabolic genes, such as nicotinamide phosphoribosyltransferase (*Nampt*), which are implicated in many aspects of metabolism and biochemical processes, therefore supporting the tight relation between the clock system and metabolism or physiology^8^,^9^. Interestingly, the expression of clock genes in the liver^10^,^11^, and in turn, metabolic circadian rhythm, is entrained by the frequency and daytime meals as well as by diet composition.

Proanthocyanidins (PAs) are a class of polyphenols present in vegetables, fruits, cacao, nuts and beverages such as red wine or tea; therefore, presence in the human diet is considerably high^12^. PAs consumption exerts a varied range of healthy effects, including the reduction of cardiovascular diseases^13^ and improvement in insulin resistance^14^, obesity^15^, inflammation^16^, hypertension^17^, oxidative stress^18^ and dyslipidemia^19^. Interestingly, the liver is a key organ in which PAs are active, restoring lipid^20^ and glucose^21^ homoeostasis after a disruption.

Remarkably, chronic PAs consumption modulates the expression of clock and clock-controlled genes in the liver, gut and white adipose tissue in healthy and obese rats^22^. However, there is little information about the effect of polyphenol consumption on circadian rhythm in the liver. Therefore, the aim of the current study was to determine whether PAs could modulate the peripheral clock system in the liver to set a new cellular mechanism by which PAs can modulate cell functionality and improve some pathological conditions. To this end, we have measured the expression rhythm of clock-core and clock-controlled genes in the liver by administering PAs during the day, at night or in jet-lagged rats.

## Results

### Acute administration of GSPE only modulated the expression pattern of clock-core and clock-controlled genes in the liver when was administered at ZT12

The capacity of GSPE to modify the molecular clock in the liver was evaluated by measuring the 24 h mRNA oscillation of *Clock* and *Bmal1* (clock core genes), *Per2* (a component of the negative loop of the circadian clock), *Rorα* and *Rev-erbα* (nuclear receptors whose expression is regulated by CLOCK:BMAL1 and which act as an activator or repressor, respectively, of Bmal1 gene expression), *Nampt* (a metabolic gene whose expression is directly regulated by CLOCK:BMAL1) and *HmgCoAR* (a metabolic gene that has circadian rhythm expression but that is not directly controlled by CLOCK:BMAL1).

**Figure 1:**
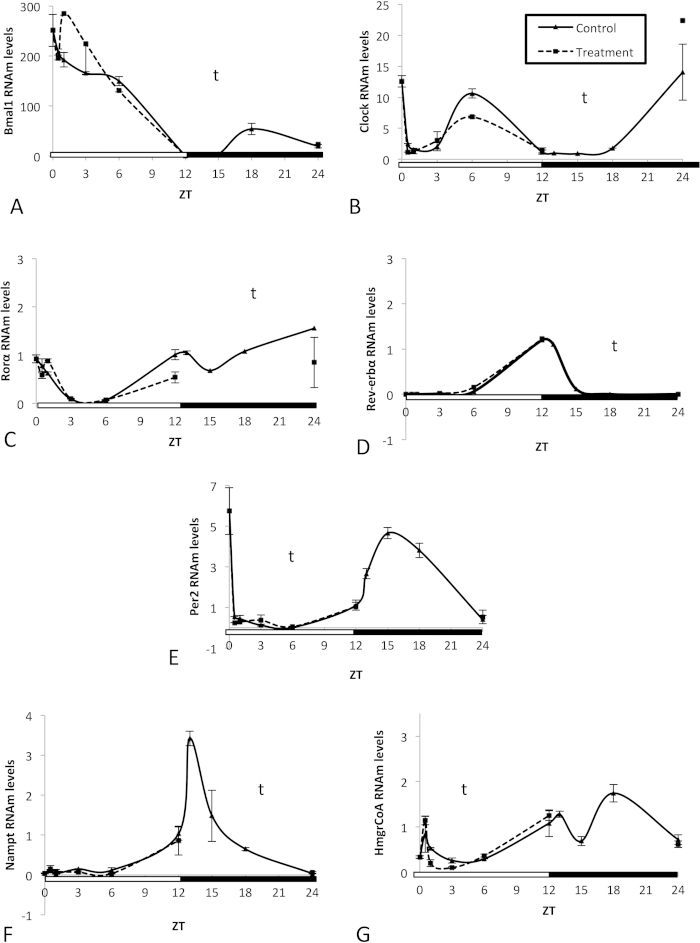
Effects of an oral dose of a grape seed proanthocyanidin extract (GSPE) administered at Zeitgeber Time 0 on clock-core and clock-controlled genes in the liver. The capacity of GSPE to modify the peripheral clock was evaluated by measuring the oscillation of the levels of mRNA from the clock core genes (**A**) *Bmal1* and (**B**) *Clock* as well as the CLOCK:BMAL1-controlled genes, (**C**) *Rorα*, (**D**) *Rev-erbα,* (**E**) *Per2* and (**F**) *Nampt.* (**G**) The expression of *HmgCoAR*, a gene that has circadian rhythm expression but that is not directly controlled by CLOCK:BMAL1, was also evaluated. Rats were orally gavaged with tap water (control group) or 250 mg of GSPE /kg body weight dissolved in tap water at ZT0 (light turned on), and rats were sacrificed at ZT0, ZT0.5, ZT1, ZT3, ZT6, ZT12 or ZT24. mRNA levels were measured by real-time qRT-PCR and their expression was normalized to cyclophilin mRNA levels. Each graph shows the mean±s.e. for each data point (n = 3). For the control group, a 24 hour curve was constructed by assembling the expression values of the control group from this experiment and those from Fig. 2. T, significant effect of proanthocyanidins; t, significant effect of Zeitgeber Time; T*t, interaction between the two variables by two-way ANOVA.

Rats were administered GSPE at ZT0 and sacrificed at ZT0, ZT0.5, ZT1, ZT3, ZT6, ZT12 or ZT24. Nonetheless, to achieve a better visualization of the changes induced by GSPE administered at ZT0, we drew the figures (Fig. 1) with a 24 h curve for the control group by assembling the expression values of the control group administered at ZT0 and ZT12. Overall, the ANOVA test indicated that GSPE administered at ZT0 (light turned on) did not significantly affect the mRNA rhythm of any gene studied in the liver (Fig. 1A–G).

**Figure 2:**
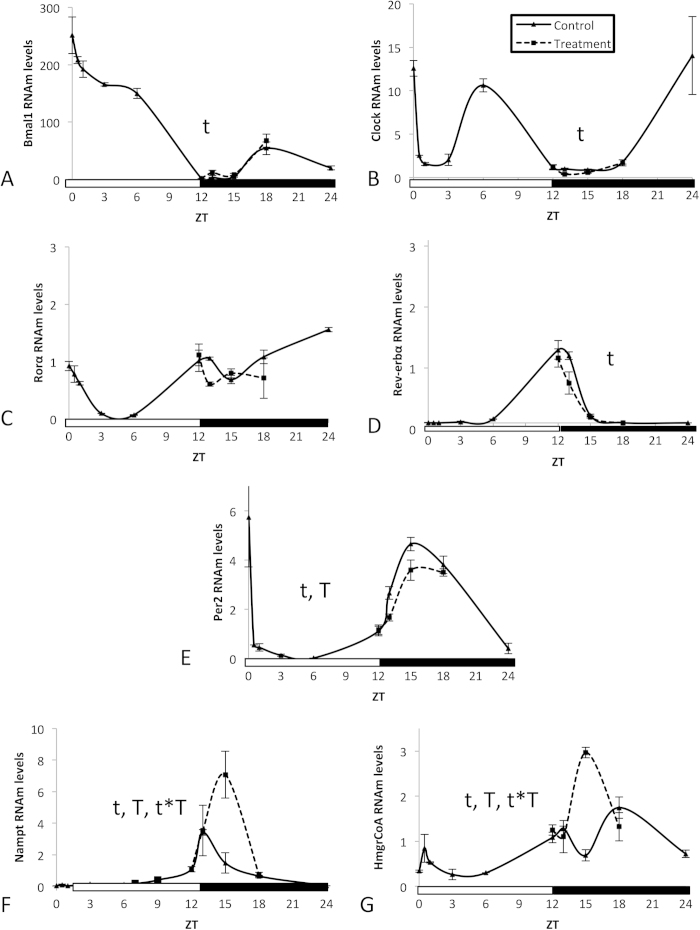
An oral dose of a grape seed proanthocyanidin extract (GSPE) administered at Zeitgeber Time 12 significantly affects the expression of Per2, Nampt and HmgCoAR in the liver. The capacity of GSPE to modify the peripheral clock was evaluated by measuring the oscillation of the levels of mRNA from the clock core genes (**A**) *Bmal1* and (**B**) *Clock* as well as the CLOCK:BMAL1-controlled genes (**C**) *Rorα*, (**D**) *Rev-erbα,* (**E**) *Per2*, and (**F**) *Nampt.* (**G**) The expression of *HmgCoAR*, a gene that has circadian rhythm expression but that is not directly controlled by CLOCK:BMAL1, was also evaluated. Rats were orally gavaged with tap water (control group) or 250 mg of GSPE /kg body weight dissolved in tap water at ZT12 (light turned off), and rats were sacrificed at ZT12, ZT13, ZT15 or ZT18. mRNA levels were measured by real-time qRT-PCR and their expression was normalized to cyclophilin mRNA levels. Each graph shows the mean±s.e. for each data point (n = 3). For the control group, a 24 hour curve was constructed by assembling the expression values of the control group from this experiment and those from Fig. 1. T, significant effect of proanthocyanidins; t, significant effect of Zeitgeber Time; T*t, interaction between the two variables by two-way ANOVA.

Rats are nocturnal animals and eat mainly at night. Therefore, we next studied whether PAs can modulate the molecular clock in the liver when GSPE is administered at night, when the liver actively manages the ingested nutrients. GSPE was administered when the light was turned off (ZT12), and rats were sacrificed at ZT12, ZT13, ZT15 or ZT18. Thus, the expression of clock-core and clock-controlled genes in the liver was determined at these four time points. Nonetheless, to achieve a better visualization of the changes induced by GSPE administered at ZT12, we drew the figures (Fig. 2) with a 24 h curve for the control group by assembling the expression values of the control group from both this experiment and the former experiment.

GSPE, administered at ZT12, induced slight effects on the mRNA levels of clock-core genes *Bmal1* (Fig. 2A) and *Clock* (Fig. 2B) as well as on the mRNA levels of the clock-controlled genes *Rorα* (Fig. 2C) and *Rev-erbα* (Fig. 2D). Nonetheless, the mRNA levels of *Per2* (Fig. 2E) and *Nampt* (Fig. 2F), two clock-controlled genes, were significantly affected by GSPE. Moreover, the mRNA levels of *HmgCoAR* (Fig. 2G), a gene with circadian rhythm but not directly controlled by the clock-core genes, were also significantly increased by GSPE treatment.

Comparing the effects of GSPE administered at ZT12 (beginning of the night) or at ZT0 (beginning of the day), it is evident that the capacity of GSPE to modulated the clock system in the liver was dependent on the time of GSPE administration, being only effective at ZT12.

### Acute administration of GSPE modulated the peripheral clock in the liver of jet-lagged rats

The capacity of GSPE to modulate the peripheral clock was also evaluated in a situation where circadian rhythm was disrupted using rats subjected to a 6 hour jet-lag. Rats at ZT6 (middle of the light period) were administered GSPE and moved to ZT12 (light turned off) and they were sacrificed at ZT12, ZT13, ZT15 or ZT18. Thus, the expression of clock-core and clock-controlled genes in the liver was determined at these four time points.

**Figure 3:**
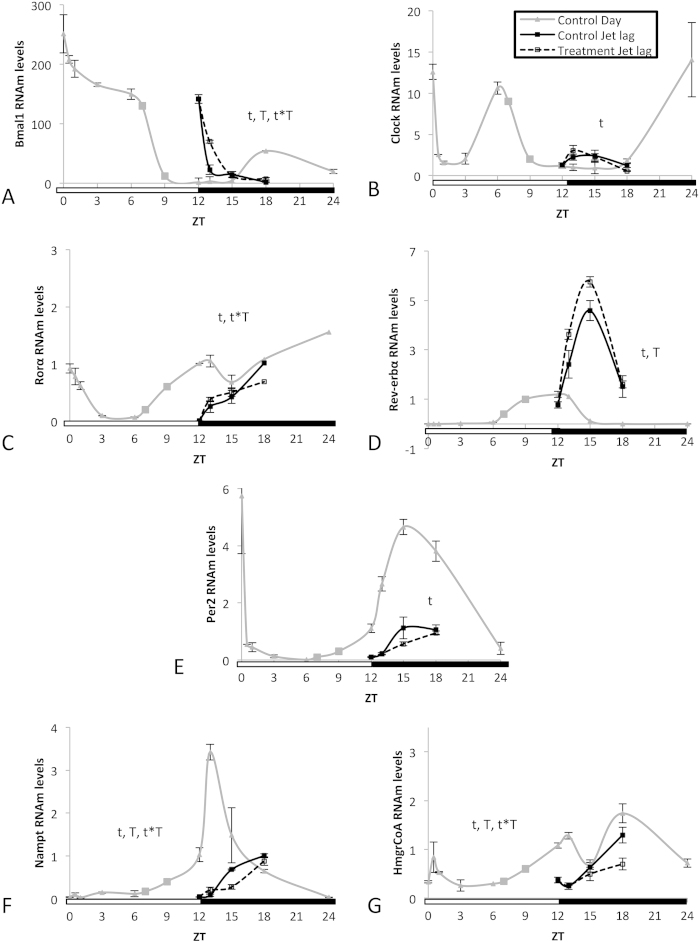
An oral dose of a grape seed proanthocyanidin extract (GSPE) administered to jet-lagged rats significantly alters the expression rhythms of *Rev-erbα, Bmal1, Nampt* and *HmgCoAR*. The capacity of GSPE to modify the peripheral clock was evaluated by measuring the oscillation of the levels of mRNA from the clock core genes (**A**) *Bmal1* and (**B**) *Clock* as well as the CLOCK:BMAL1 controlled genes (**C**) *Rorα*, (**D**) *Rev-erbα,* (**E**) *Per2* and (**F**) *Nampt.* (**G**) The expression of *HmgCoAR*, a gene that has circadian rhythm expression but that is not directly controlled by CLOCK:BMAL1, was also evaluated. An oral dose of a grape seed proanthocyanidin extract (GSPE) administered to jet-lagged rats significantly alters the expression rhythms of *Rev-erbα*, *Bmal1*, *Nampt* and *HmgCoAR*. Rats were orally gavaged with tap water (control group) or 250 mg of GSPE /kg body weight dissolved in tap water at ZT6 (middle of the light period) and were moved to ZT12 (light turned off). Rats were sacrificed at ZT12, ZT13, ZT15 or ZT18. mRNA levels were measured by real-time qRT-PCR and their expression was normalized to cyclophilin mRNA levels. Each graph shows the mean±s.e. for each data point (n = 3). For the control group with no jet lag, a 24 hour curve was constructed by assembling the expression values of the control groups from Figs. 1 and 2. T, significant effect of proanthocyanidins; t, significant effect of Zeitgeber Time; T*t, interaction between the two variables by two-way ANOVA.

In control animals, the jet lag induced a clear shift in the mRNA rhythmicity for all of the genes that were studied (Fig. 3) when the rhythms were compared with the 24 h control waves (built by assembling the expression values of the control groups, such as in the former experiments).

GSPE, administered at the beginning of jet lag, did not modulate *Clock* (Fig. 3B) or *Per2* (Fig. 3E), whereas it significantly altered the expression rhythm of *Rev-erbα* (Fig. 3D), *Bmal1* (Fig. 3A), *Nampt* (Fig. 3F) and *HmgCoAR* (Fig. 3G) when compared with the jet lag control group. Remarkably, *Nampt* and *HmgCoAR* were again two of the genes most sensitive to GSPE, as in when GSPE was administered at ZT12. Thus, next we focused in these two genes.

### Acute administration of GSPE had opposite effects on both Nampt expression and NAD levels in the liver at different treatment times

Given that *Nampt* gene was susceptible to GSPE in the liver and that this protein is the rate-limiting enzyme of the NAD salvage pathway, we next focused specifically on the oscillations of *Nampt* RNAm and protein levels as well as NAD concentration during the first 6 hours after GSPE administration at ZT0 and ZT12. These analyses were performed using the livers of rats from the previous experiments.

**Figure 4:**
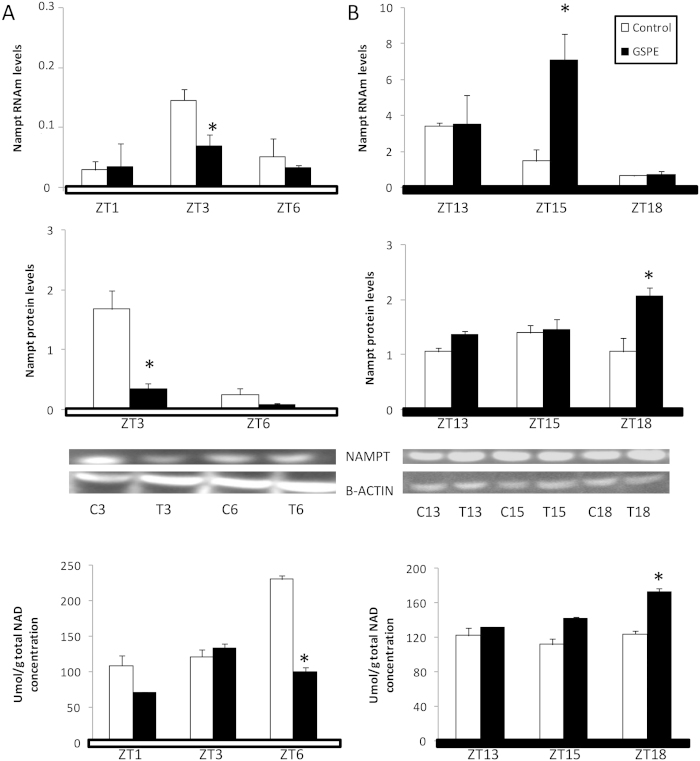
An oral dose of a grape seed proanthocyanidin extract (GSPE) administered at Zeitgeber Time 0 or 12 oppositely affects both Nampt expression and NAD levels in the liver. Rats were orally gavaged with tap water (control group) or 250 mg of GSPE / kg body weight dissolved in tap water both at (**A**) ZT0 (light turned on) and (**B**) ZT12 (light turned off), and rats were sacrificed at ZT1, ZT3 or ZT6 and ZT13, ZT15 or ZT18, respectively. Nampt RNAm levels were measured by real-time qRT-PCR and its expression was normalized to cyclophilin mRNA levels. Nampt protein was analyzed by Western blot and was normalized to β-actin. Un-cropped blots/gels are presented in Supplementary Figure 1. NAD quantification was performed using an ELISA kit following the manufacturer’s instructions. Each value is the mean ± s.e. of the same 3 animals for all the parameters quantified. White bars, control group; colored bars, GSPE-treated groups. *Statically significant differences found by independent Student T-test (p < 0.05) between the control group and GSPE-treated group for each ZT.

Remarkably, GSPE induced opposite effects when it was administered at ZT0 or ZT12 (Fig. 4). *Nampt* protein and mRNA levels were decreased three hours after GSPE administration at ZT0 (Fig. 4A), while *Nampt* mRNA and protein levels were significantly elevated at three and six hours, respectively, after GSPE administration at ZT12 (Fig. 4B). These modifications in *Nampt* expression agreed with the alterations in NAD levels that were induced by GSPE in each situation: NAD levels were significantly decreased six hours after GSPE administration at ZT0, whereas they were significantly increased six hours after GSPE administration at ZT12.

Altogether, these results implicate *Nampt* and NAD modulation as key factors in GSPE activity in the liver and confirm *Nampt* as a target of GSPE in the liver

### Acute administration of GSPE at ZT12 increased the ratio of acetylated Bmal1 in rat liver

*Nampt* is a direct target gene of CLOCK:BMAL1. However, GSPE did not induce a strong modification in the mRNA rhythms of *Clock* or *Bmal1* that could explain the observed alteration in *Nampt* expression. However, the transcriptional activity of CLOCK:BMAL1 is dependent on *Bmal1* acetylation. Thus, we focused further on the ratio of *Bmal1* acetylation by measuring *Bmal1* mRNA, protein and acetylated protein during the first six hours after GSPE administration at ZT0 or ZT12. *Bmal1* mRNA, protein and acetylated protein were analyzed in the livers of rats from the previous experiments.

**Figure 5:**
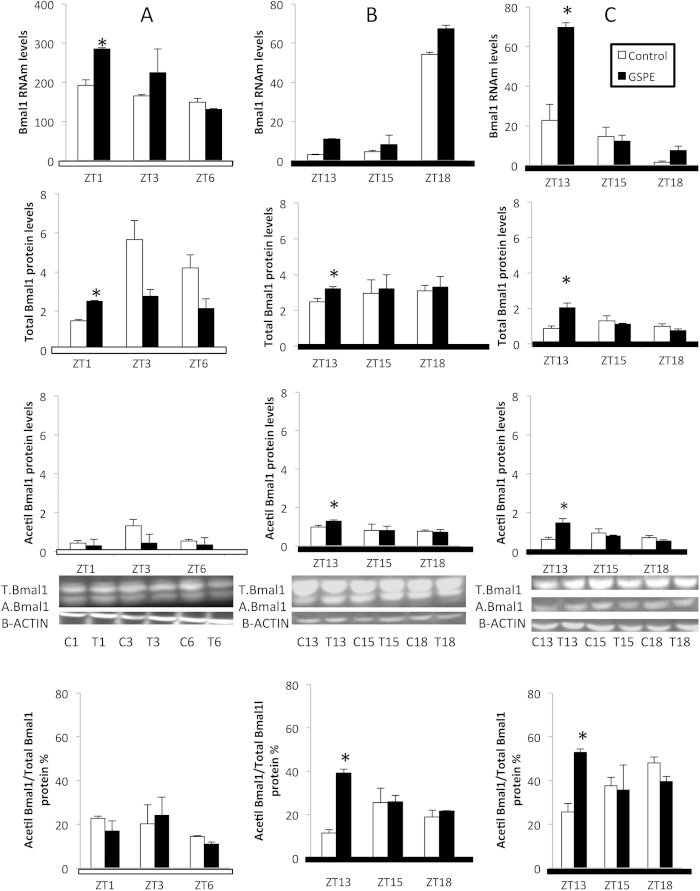
An oral dose of a grape seed proanthocyanidin extract (GSPE) administered at Zeitgeber Time 12 or to jet-lagged rats increases the ratio of acetylated Bmal1 in rat liver. Rats were orally gavaged with tap water (control group) or 250 mg of GSPE /kg body weight dissolved in tap water both at (**A**) ZT0 (light turned on), (**B**) ZT12 (light turned off) or (**C**) ZT6 (middle of the light period), in the case of the jet-lagged rats, which were moved to ZT12 (light turned off) after GSPE administration. Rats were sacrificed at ZT1, ZT3 or ZT6 when GSPE was administered at ZT0 and ZT13, ZT15 or ZT18 when GSPE was administered at ZT12 or to jet-lagged rats. Bmal1 RNAm levels were measured by real-time qRT-PCR and its expression was normalized to cyclophilin mRNA levels. Acetylated and total BMAL1 proteins was analyzed by Western blot and was normalized to β-actin. Acetylated and total BMAL1 protein samples were then divided to obtain the acetylated Bmal1/total Bmal1 protein ratio, shown as a percentage. Un-cropped blots/gels are presented in Supplementary Figure 1. Each value is the mean±s.e. of the same 3 animals for all the parameters quantified. White bars, control group; colored bars, GSPE-treated groups. *Statically significant differences found by independent Student T-test (p < 0.05) between the control group and GSPE-treated group for each ZT.

GSPE administered at either time increased mRNA and protein levels of *Bmal1* after 1 hour of treatment (Fig. 5A,B; ZT1 and ZT13, respectively). Nonetheless, the ratio of acetylated *Bmal1* was only increased when GSPE was administered at night (ZT12); these are the same circumstances under which *Nampt* was overexpressed.

### Acute administration of GSPE repressed the relative expression of HmgcoAR in rat liver

Finally, the mRNA and protein levels of *HmgcoAR,* a metabolic gene not controlled by the clock molecular machinery, were determined according to the three experimental designs (day, night and jet lag) during only the first six hours after GSPE treatment (ZT1, ZT3 and ZT6 or ZT13, ZT15 and ZT18). *HmgcoAR* mRNA and protein were analyzed in the livers of rats from the previous experiments.

**Figure 6:**
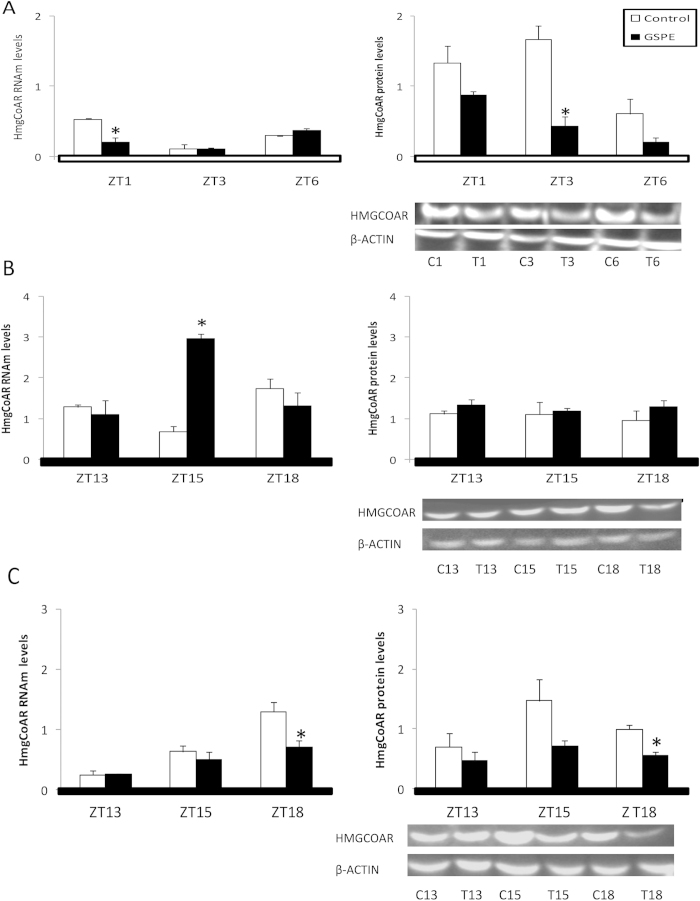
An oral dose of a grape seed proanthocyanidin extract (GSPE), administered at Zeitgeber Time 0, 12, or to jet-lagged rats, modulates HmgcoAR expression in rat liver. Rats were orally gavaged with tap water (control group) or 250 mg of GSPE / kg body weight dissolved in tap water at (**A**) ZT0 (light turned on), (**B**) ZT12 (light turned off) or (**C**) ZT6 (middle of the light period), in the case of the jet-lagged rats, which were moved to ZT12 (light turned off) after GSPE administration. Rats were sacrificed at ZT1, ZT3 or ZT6 when GSPE was administered at ZT0 and ZT13, ZT15 or ZT18 when GSPE was administered at ZT12 or to jet-lagged rats. HmgcoAR RNAm levels were measured by real-time qRT-PCR and its expression was normalized to cyclophilin mRNA levels. HmgcoAR protein was analyzed by Western blot and was normalized to β-actin. Un-cropped blots/gels are presented in Supplementary Figure 1. White bars, control group; colored bars, GSPE-treated groups. *Statically significant differences found by independent Student T-test (p < 0.05), between the control group and GSPE-treated group for each ZT.

*HmgcoAR* expression was significantly repressed at ZT1 (mRNA) and ZT3 (protein) after GSPE treatment was administered at ZT0 (Fig. 6A). While mRNA levels were increased at ZT15 after GSPE treatment was administered at ZT12, the protein levels did not reflect that fact (Fig. 6B). Finally, in the jet-lagged rats, *HmgcoAR* protein and mRNA levels were decreased at ZT18 (Fig. 6C).

## Discussion

While light is the major synchronizer of the central clock in the suprachiasmatic nucleus (SCN) in the hypothalamus, many other external cues such as temperature, social events or meal timing^10^ can entrain circadian rhythms in other cerebral regions or peripheral tissues. This phenomenon is especially the case in the liver, which is the most important metabolic organ due to its involvement in glucose^6^ and lipid^5^ metabolism, among other crucial physiological functions^23^. Even specific components in foods could also be important synchronizers, such as dietary fat^24^ or phenolic compounds like resveratrol^25^,^26^,^27^,^28^. Therefore, the aim of this work was to determine the capacity of an acute dose of GSPE to act as a signal to modulate the molecular clock in the liver.

To accomplish this outcome, three different experimental approaches were performed to determine whether PAs can modulate the liver clock: the administration of GSPE at ZT0, at the beginning of the light phase; at ZT12, at the beginning of the dusk phase; and to rats with 6 h of jet lag. The data clearly show that the power of PAs to modulate the circadian rhythm of clock-core and clock-controlled genes in the liver depends on the time of their administration.

Interestingly, *Nampt* and NAD emerge as molecular targets of PAs in the liver. *Nampt* is the rate-limiting enzyme in NAD biosynthesis through its salvage pathway^29^. NAD plays a major role as a coenzyme in numerous oxidation-reduction reactions^30^ and is required in a number of important signaling pathways in mammalian cells, including poly-ADP-ribosylation in DNA repair^31^, mono-ADP-ribosylation in both the immune response and G protein-coupled signaling^32^, and the synthesis of cyclic ADP-ribose and nicotinate adenine dinucleotide phosphate (NAADP) in intracellular calcium signaling^33^. Furthermore, NAD activates several NAD(+)-dependent deacetylases (SIRT), such as SIRT1 and SIRT3, thus controlling the activity of many cellular proteins by cycling them between their acetylated and deacetylated forms. Specifically, the circadian oscillations of NAD levels have been shown to modulate mitochondrial respiration by controlling the activity of SIRT3, thus generating rhythms in the acetylation and activity of oxidative enzymes that synchronize mitochondrial oxidative functions across the daily cycles of fasting and feeding^34^. Therefore, NAD is a key molecule in the synchronization of liver metabolism, and the modulation of its levels in the liver by PAs is an attractive candidate for the explanation of some of the metabolic effects of PAs. GSPE modulated *Nampt* and NAD levels in opposite ways 6 h after its administration during the day versus at night, reducing or increasing their levels, respectively, suggesting that *Nampt* expression and NAD levels peaked at night. Therefore, in this sense, PAs could act as an element of adaptation in the liver, improving the energetic profile of rats and increasing mitochondrial function and oxidation at night, when rats are active. In keeping with the idea of an adaptation mechanism, as these animals are resting during the light phase, PA activity could be acting as an energy saver through the decreased levels of NAD after PA administration at ZT0.

NAD concentration oscillates in a circadian manner due to the circadian expression of *Nampt*, which in turn is mediated by the CLOCK:BMAL1 heterodimer^35^. The rhythm of *Clock* and *Bmal1* expression was not altered by GSPE administration, either during the day or at night (Figs. 1 and 2). However, when *Bmal1* expression was studied during the first six hours of GSPE administration (Fig. 5), both mRNA and protein levels were always increased one hour after PA consumption, regardless of whether GSPE was administered diurnally, at night, or even under jet lag conditions, suggesting a robust relationship between *Bmal1* and PAs. However, to be active, BMAL1 should be acetylated by CLOCK, which is its own partner. Therefore, the ratio of acetylated to total BMAL1 protein provides direct information about the transactivation activity of BMAL1:CLOCK^35^. We found that GSPE significantly increased the ratio of BMAL1 that was acetylated at ZT13, whereas this effect was not observed at ZT1. This differential pattern of BMAL1 acetylation, which depends on the time of GSPE administration, could explain the overexpression of NAMPT and therefore the peak in NAD levels in the liver only when GSPE was administered at night. Therefore, it is globally supposed in this work that NAD levels peaked 6 hours after GSPE consumption at ZT12 (thereby at ZT18) as a consequence of an increased BMAL1 acetylation ratio at ZT13 that, in turn, increased *Nampt* mRNA and protein levels at ZT15-ZT18.

PAs modulate lipid metabolism in the liver^20^. Therefore, we also analyzed the expression of *HmgcoAR*, the key enzyme in the cholesterol biosynthetic pathway, which has circadian rhythm but is not directly controlled by the clock-core genes^36^. GSPE had a dual effect on *HmgcoAR* expression depending on the time of its administration. GSPE repressed both *HmgcoAR* mRNA and protein levels at ZT0, whereas protein levels at ZT12 were not affected. Therefore, as in the case of BMAL1 acetylation, Nampt expression and NAD levels, the time of PA administration conditions the circadian regulation outputs.

Further studies are needed in order to find out the actual compounds that can modulate the molecular clock in the liver. Studies on the bioavailability of proanthocyanidins are controversial^37^. Some studies indicate no absorption of oligomeric proanthocyanidins, thus attributing the biological effects of PA to their colonic metabolites such as phenolic acids and valerolactones^37^. However, others studies have detected oligomeric PA in plasma^38^ and in several tissues^39^ of rats. Moreover, GSPE can be metabolized by intestinal and hepatic enzymes, producing a large array of metabolites^39^.

Overall, these findings agree with the Xenohormesis Hypothesis, which proposes that heterotrophs are able to sense chemical cues, such as polyphenols, that are synthesized by plants in response to stress^40^. In fact, circadian rhythms allow the anticipation of environmental changes and adaptation to the time of day and food availability, which has been shown in this work through NAD, NAMPT and BMAL1 acetylation levels. Thus, PAs can advise animals about environmental conditions by modulating biological rhythms to obtain a better ability to adapt to changing conditions over the course of their lives. Despite this study has been performed with a pharmacological dose of PAs, animals could consume such a huge amount of PAs in a wildlife situation.

In conclusion, PAs modulate the molecular clock in the liver even though their effectiveness depends largely on the time of administration. Specifically, *Bmal1* and *Nampt,* as well NAD, emerge as targets of GSPE in the liver.

## Materials and Methods

### Grape seed proanthocyanidin extract composition

Grape seed proanthocyanidin extract (GSPE) was kindly provided by Les Dérives Résiniques et Terpéniques (Dax, France). Specifically, GSPE contains^41^: catechin (58 μmol/g), epicatechin (52 μmol/g), epigallocatechin (5.50 μmol/g), epicatechingallate (89 μmol/g), epigallocatechingallate (1.40 μmol/g), dimericprocyanidins (250 μmol/g), trimericprocyanidins (1568 μmol/g), tetramericprocyanidins (8.8 μmol/g), pentamericprocyanidins (0.73 μmol/g) and hexamericprocyanidins (0.38 μmol/g).

### Animals

All procedures involving the use and care of animals were reviewed and approved by The Animal Ethics Committee of the Universitat Rovira i Virgili (Permit number 4249 by Generalitat de Catalunya). All experiments were performed in accordance with relevant guidelines and regulations.

Eighty-four eight-week-old male Wistar rats (Crl: WI (Han)) were purchased from Charles River (Barcelona, Spain) and fed ad libitum with a standard chow diet (STD, Panlab 04, Barcelona, Spain) and tap water. Rats were divided into three groups according to the Zeitgeber time (ZT) when GSPE was administered.

#### Administration of GSPE at ZT0

Forty rats were singly caged in animal quarters at 22 °C with a 12 h light/dark cycle (light from 9:00 to 21:00 pm). After three weeks of adaptation, the rats were orally gavaged with tap water (control group) or 250 mg of GSPE/kg body weight dissolved in tap water at ZT0 (9:00 am, light turned on). Rats were sacrificed by beheading at ZT0, ZT0.5, ZT1, ZT3, ZT6, ZT12 and ZT24 (n = 3 for control and n = 3 for GSPE-treated groups).

#### Administration of GSPE at ZT12

Twenty-two rats were singly caged in animal quarters at 22 °C with a 12 h light/dark cycle (light from 21:00 pm to 9:00 am). After three weeks of adaptation, the rats were orally gavaged with tap water (control group) or 250 mg of GSPE/kg body weight dissolved in tap water at ZT12 (9:00 am, light off). Rats were sacrificed by beheading at ZT12, ZT13, ZT15, and ZT18 (n = 3 for control and n = 3 for GSPE-treated groups).

#### Administration of GSPE to jet-lagged rats

Twenty-two rats were singly caged in animal quarters at 22 °C with a 12 h light/dark cycle (light from 15:00 pm to 03:00 am). After three weeks of adaptation, rats were orally gavaged with tap water (control group) or 250 mg of GSPE /kg body weight dissolved in tap water at ZT6 (9:00 am, middle of the light day) and immediately moved to a dusk room (ZT12), thus giving rats a jet lag of 6 hours. Rats were sacrificed by beheading at ZT12, ZT13, ZT15, and ZT18 (n = 3 for control and n = 3 for GSPE-treated groups).

For the three experiments, the liver was excised, frozen immediately in liquid nitrogen and stored at −80 °C until RNA and protein extraction.

### RNA extraction and cDNA synthesis

Total RNA from liver was extracted using TRIzol reagent and an RNeasy Mini Kit (Qiagen, 74106, Barcelona, Spain) according to manufacturer protocols. RNA was quantified by spectrophotometry (Nanodrop 1000 Spectrophotometer, Thermo Scientific, Madrid, Spain) at *λ* = 260 nm and tested for purity (by A260/280 ratio) and integrity (by denaturing gel electrophoresis). Complementary DNA was generated using the High-Capacity complementary DNA Reverse Transcription Kit from Applied Biosystems (4368814, Madrid, Spain).

### mRNA quantification by real-time qRT-PCR

A total of 10 ng of cDNA was subjected to quantitative RT-PCR amplification using SYBR Green PCR Master Mix from Bio-Rad (172-5200, Barcelona, Spain). The forward and reverse primers of the genes analyzed are shown in Table 1. Reactions were run on a quantitative real-time PCR system (CFX96 touch of Bio-Rad, Barcelona, Spain); the thermal profile settings were 50 °C for 2 min, 95 °C for 2 min, and then 40 cycles at 95 °C for 15 s and 60 °C for 2 min. Finally, statistical data were converted and normalized to the linear form by the 2^−^CT (∆∆C_T_) calculation^42^. The relative expression of the clock genes was normalized to cyclophilin mRNA levels.

### Western blot analyses

Protein was extracted from liver using RIPA (radio-immunoprecipitation assay) lysis buffer (15 mM Tris–HCl, 165 mM NaCl, 0.5% Na-deoxycholate, 1% Triton X-100 and 0.1% SDS) containing a protease inhibitor cocktail (1:1000; Sigma-Aldrich P8340-1 mL, Madrid, Spain) and 1 mM PMSF (phenylmethanesulfonyl fluoride solution, Sigma-Aldrich 93482, Madrid, Spain). The total protein levels of the lysates were determined using the BCA method from Thermo Scientific (23227, Barcelona, Spain). The samples were then placed in sample buffer (0.5 M Tris–HCl, pH 6.8; 10% glycerol; 2% (w/v) SDS; 5% (v/v) β-mercaptoethanol; and 0.05% bromophenol blue). After boiling for 5 min, 50 μg of protein was loaded and separated on a 10% SDS-polyacrylamide gel. The samples were then transferred to a polyvinylidene fluoride (PVDF) membrane (Bio-Rad Laboratories, 162-017, Barcelona, Spain) using a transblot apparatus (Bio-Rad, 16580229SP ) and blocked at room temperature for 1 h with 5% (w/v) non-fat milk in TTBS buffer (Tris-buffered saline (TBS) plus 0.5% (v/v) Tween-20). The membranes were incubated overnight at 4 °C with primary monoclonal antibodies directed against Nampt (Imgenex, IMX-6096, Nanterre, France), Bmal1 (LS-Bio, LS-C16603, Vizcaya, Spain), acetyl-Bmal1 (Millipore, AB15396, Madrid, Spain), HmgcoAR (Santa Cruz, SC-33827, Nanterre, France) and anti-β-actin (Sigma-Aldrich, A2066-0.2 mL, Madrid, Spain) at a 1:1000 dilution in blocking solution. After washing with TTBS, the blots were incubated with a peroxidase-conjugated monoclonal anti-rabbit secondary antibody (Sigma-Aldrich, A1949, Madrid, Spain) at a 1:10,000 dilution at room temperature for 1.5 h. The blots were then washed thoroughly in TTBS followed by TBS. Immunoreactive proteins were visualized with an enhanced chemiluminescence substrate kit (ECL plus; Amersham Biosciences, GE Healthcare, RPN2132, Barcelona, Spain) according to the manufacturer’s instructions. Images were obtained with a GBOX Chemi XL 1.4 image system (Syngene, UK). Band quantification was performed with ImageJ software (NIH, USA). The results were expressed as relative intensity (Nampt/ β-actin, Bmal1/ β-actin, HmgcoAR/ β-actin and acetyl-Bmal1/ β-actin) and are relative to the loading control group.

### NAD quantification

NAD levels in the liver were quantified using an ELISA kit following the manufacturer’s instructions (Sigma-Aldrich, MAK037-1KT, Madrid, Spain).

### Data and statistical analysis

The results are presented as the mean with the associated standard error (SE).The data were analyzed using a two-way ANOVA and Student t-test to determine the significant difference using SPSS statistical software (version 17.0 for Windows; SPSS, Inc.). P values < 0.05 were considered statistically significant.

## Additional Information

**How to cite this article**: Ribas-Latre, A. *et al.* Dietary proanthocyanidins modulate BMAL1 acetylation, Nampt expression and NAD levels in rat liver. *Sci. Rep.* **5**, 10954; doi: 10.1038/srep10954 (2015).

## Supplementary Material

Supplementary Information

## Figures and Tables

**Table 1:**
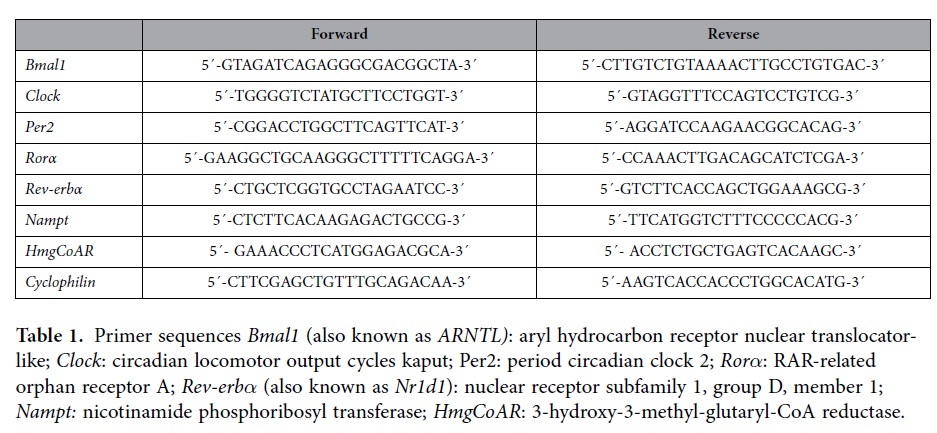
Primer sequences
