# Supplementary material for: Dietary proanthocyanidins modulate BMAL1 acetylation, Nampt expression and NAD levels in rat liver
Source: Sci Rep. 2015 Jun 8;5:10954. doi: 10.1038/srep10954 (PMC4603780; doi:10.1038/srep10954)

Manuscript SREP-14-11672

Dietary proanthocyanidins modulate BMAL1 acetylation, Nampt expression and NAD levels in rat liver

Aleix Ribas-Latre, Laura Baselga-Escudero, Ester Casanova, Anna Arola-Arnal, M-Josepa Salvadó, Cinta Bladé*, Lluís Arola

Supplementary Figure 1

Representative un-cropped blots/gels of Bmal1, acetylated Bmal1, HmgcoAR and Nampt

BMAL1 (69kDa)

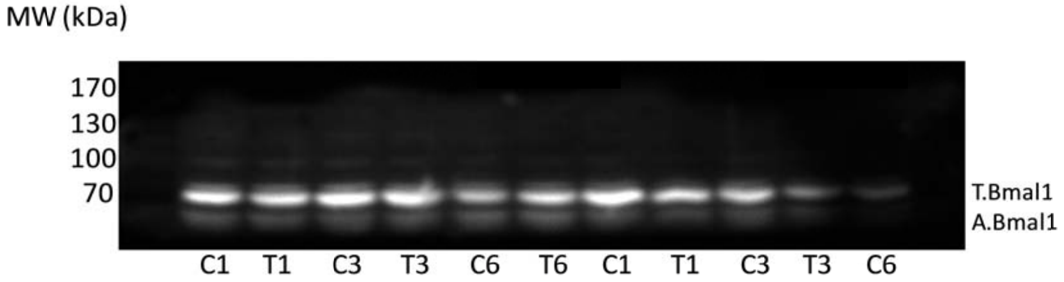

HmgCoAR (100kDa)

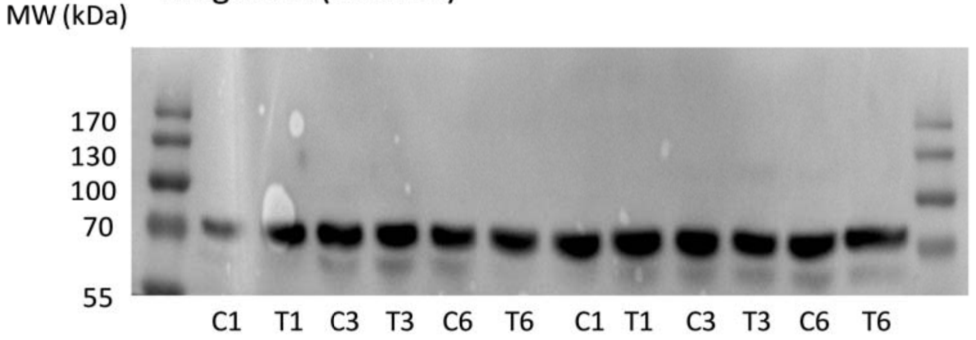

NAMPT (36kDa)

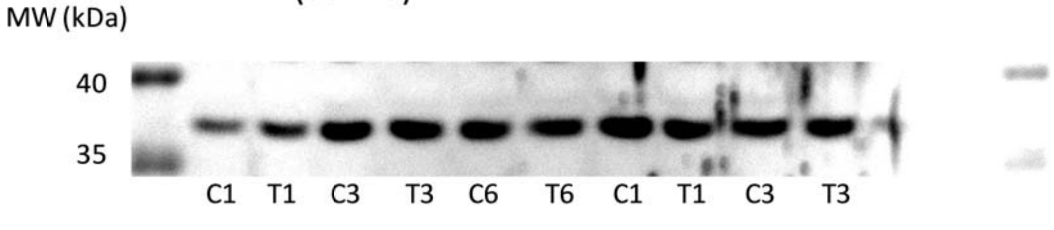

Supplement: Supplementary Information [file srep10954-s1.pdf]
